# The Use of Cone Beam Computed Tomography in Assessing the Insertion of Bone Conduction Hearing Implants

**DOI:** 10.3389/fsurg.2017.00038

**Published:** 2017-07-24

**Authors:** Tim George Ate Calon, Martin Lars Johansson, Elske Larissa van den Burg, Anna Maria Louisa Janssen, Marc van Hoof, Robert Jan Stokroos

**Affiliations:** ^1^Department of Otorhinolaryngology, Head and Neck Surgery, Maastricht University Medical Center, Maastricht, Netherlands; ^2^Oticon Medical AB, Askim, Sweden; ^3^Department of Biomaterials, Institute of Clinical Sciences, Sahlgrenska Academy, University of Gothenburg, Gothenburg, Sweden; ^4^Department of Methodology and Statistics, Care and Public Health Research Institute, Maastricht University, Maastricht, Netherlands

**Keywords:** bone-anchored hearing implants, radiology, BAHA, Osstell, implant stability, resonance frequency analysis

## Abstract

**Objective:**

This study aimed to compare postoperative cone beam CT (CBCT) imaging to implant stability quotient (ISQ) measurement and direct caliper measurements as a suitable technique to assess bone conduction hearing implant (BCHI) seating and insertion depth.

**Methods:**

*In vitro*, BCHIs were completely (*n* = 9) and partially inserted (*n* = 9) in bone blocks of different densities and subsequently scanned. Scans were processed using 3DSlicer 4.3.1 and Mathematica 10.3. ISQ measurements were obtained for all BCHIs mounted with different abutment lengths (9, 12, and 14 mm). CBCT imaging was performed for patients with a clinical indication.

**Results:**

*In vitro*, 95% prediction intervals for partially inserted and completely inserted BCHIs were determined. ISQ values significantly decreased with partial insertion, low-density artificial bone, and longer abutment lengths. Evaluation of *in vitro* and *in vivo* 3D models allowed for assessment of insertion depth and inclination.

**Conclusion:**

CBCT imaging allows to study implant seating and insertion depth after BCHI surgery. This can be useful when visual confirmation is limited. It is possible to distinguish a partial BCHI insertion from a complete insertion in artificial bone blocks. This technique could prove to be a valuable research tool. *In vitro*, ISQ values for Ponto BCHIs relate to abutment length, insertion depth, and artificial bone density.

## Introduction

In recent years, surgical placement of bone conduction hearing implants (BCHIs) has become less invasive with the introduction of the linear incision technique with tissue preservation (1). In an attempt to further improve outcomes, punch only techniques have been described with good initial results (2–4). In line with these developments, the punch only minimally invasive Ponto surgery (MIPS) technique was recently introduced (5, 6) to standardize this procedure.

During BCHI surgery, the implant should be placed perpendicular to the skull to allow for full and straight insertion. Using conventional techniques, the bone bed is visible which allows for visual feedback regarding insertion depth and implant angle. However, with tissue preservation, surgical technique visibility is reduced and with punch only techniques visual confirmation is even further obstructed by the surrounding tissue. In absence of visual confirmation, we sought to obtain an objective feedback tool to verify complete insertion and angulation.

Imaging techniques might provide objective feedback regarding insertion depth and angulation of BCHI seating. However, traditional plain radiography techniques cannot show the necessary 3D detail and conventional CT imaging is difficult to justify due to its radiation burden and scattering sensitivity. Cone beam CT (CBCT) is characterized by high resolution, reduced sensitivity to scattering artifacts, lower overall radiation doses compared to conventional CT imaging. CBCT imaging has been described as an appropriate technique for the evaluation of peri-implant bone for dental implants and cochlear implant position (7–10).

Another possibility to assess implant insertion is the Implant Stability Quotient (ISQ), a non-invasive method based on resonance frequency analysis (11). This method is being propagated as a method to assess implant stability in temporal bones (12). ISQ measurements can be obtained during surgery, possibly allowing direct intraoperative feedback and intervention. Although ISQ values are regularly reported in BCHI studies (12–14), a high level of uncertainty surrounds the clinical utility of ISQ for BCHIs. Limited consistent information is available on the multivariable interplay of clinically relevant or irrelevant factors for BCHIs. Factors described in dental studies include drilling protocol, abutment length, abutment morphology, abutment weight, implant design and surface morphology, bone density, bone to implant contact, and most importantly: osseointegration (15). Moreover, how and to what extent, ISQ measurements are affected by incomplete or angulated insertion is unknown. Before ISQ measurements can be used as a clinical diagnostic, studies proving unambiguous interpretation and validity are needed. At the moment, these are not available.

This explorative study aimed to investigate if postoperative CBCT imaging is a suitable technique to assess BCHI seating and insertion depth either *in vitro* or *in vivo*. *In vitro* validation of this technique was done in an experimental setting. In the clinical part of this study, CBCT imaging of the BCHI was performed in patients when the surgeon was uncertain about either insertion depth or insertion angle. To interpret *in vivo* seating, we matched these retrospectively to our *in vitro* results. Additionally, it was determined how ISQ values are affected by degree of insertion, abutment length and artificial bone density *in vitro*.

## Materials and Methods

### Ethics

The procedures in this study were in accordance with legislation (the Medical Research Involving Human Subjects Act) and ethical standards on human experimentation in the Netherlands. CBCT scans were made to assess BCHI seating on clinical indication. According to the Medical Research Involving Human Subjects Act, ethical approval was not required due to the nature and anonymization of the data.

### Imaging Analysis

All CBCT scans were acquired using the I-CAT scanner (Imaging Sciences International, Hatfield, PA, USA) with 0.125 mm isometric resolution. Tube current was 37.07 mAs with a tube voltage of 120 Kv. A full rotation took 26.7 s. Scans were processed with 3D Slicer 4.3.1 (http://slicer.org). A region of interest was selected, containing the BCHI and (artificial) bone adjacent to the BCHI. Fixed threshold gray-level values were used to create segmented volumes of the BCHI (artificial or real) bone and soft tissue. The segmented volumes were imported in Mathematica 10.3 (Wolfram Research, Champaign, IL, USA) to create 3D models.

### *In Vitro* Validation

#### Artificial Bone Blocks

**Figure 1:**
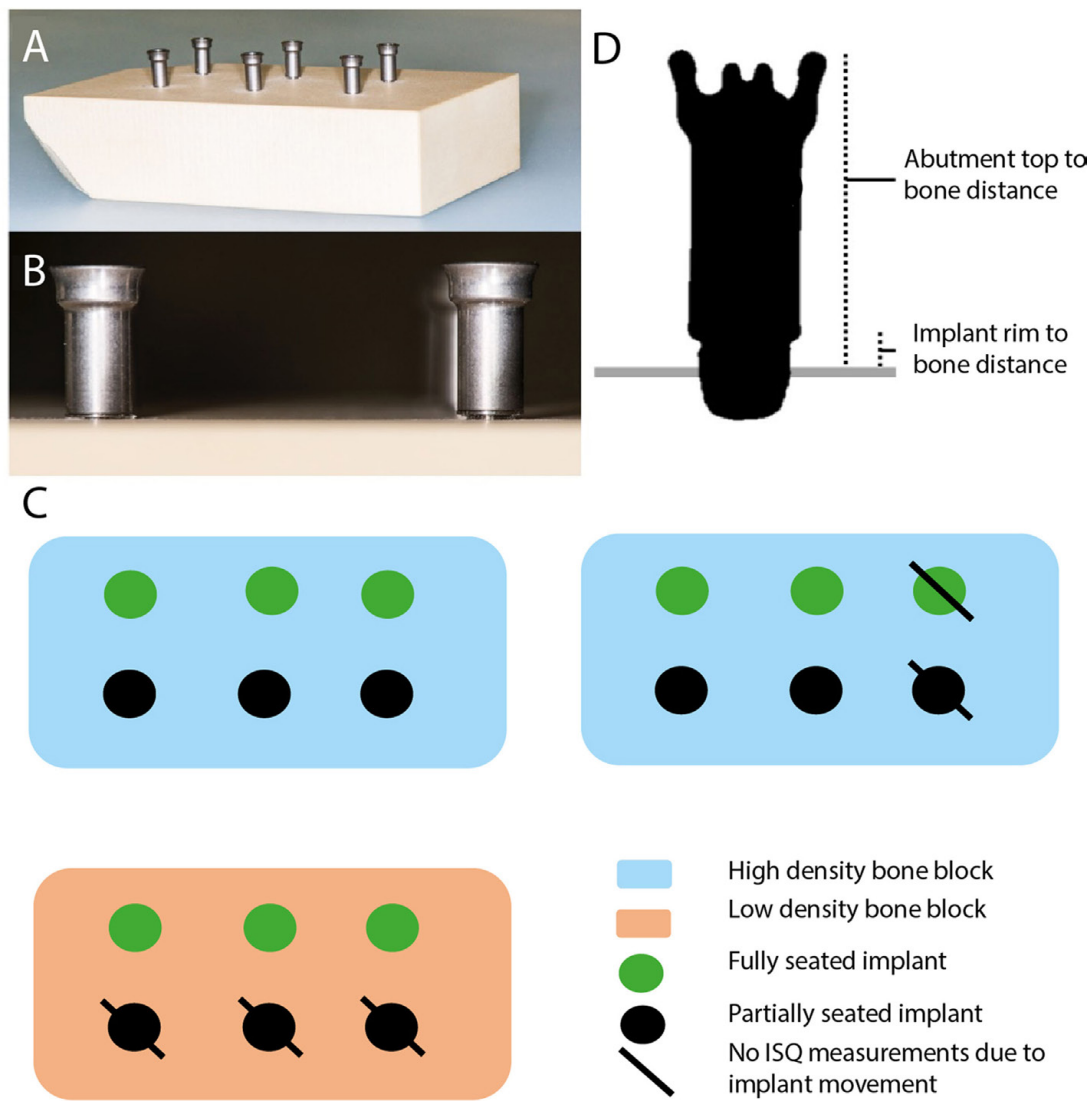
Overview of the system and the measurement points. **(A)** Overview of artificial bone block with installed implant. **(B)** Overview of fully seated bone conduction hearing implant (BCHI) (left) and partially seated BCHI (right). **(C)** Overview of artificial bone blocks. BCHI insertion measurements were performed on all bone blocks. \ indicates implants not used for implant stability quotient (ISQ) measurements due to gross implant mobility. **(D)** Coronal cross section plane of the BCHI. Measurement points are placed at the abutment top, bottom of the implant rim, and (artificial) bone at four equally spaced quadrants. In this exemplary case, the implant is partially seated.

Bone conduction hearing implants were installed in polyurethane artificial bone blocks (13 cm × 8.8 cm × 4 cm) with different densities (Sawbones, USA) (Figure 1A). Two high-density (50 pounds per cubic foot) and one low-density (40 pounds per cubic foot) artificial bone blocks were created. Installation of 4 mm wide implants (Oticon Medical AB, Askim, Sweden) mounted with 14 mm abutments (Oticon Medical AB, Askim, Sweden) were carried out using the surgical instrument designed for MIPS (5). Implants were fully inserted with 4.5 rotations or partially inserted with 3.5 rotations (Figure 1B).

#### BCHI Insertion Measurements

Virtual insertion depth measurements on the CBCT and direct manual caliper measurements were completed for 18 implants placed in three bone blocks (Figure 1C). According to specifications provided by the manufacturer, the distance from the top of the abutment (14 mm) and the implant rim should range between 14.43 and 14.59 mm when fully inserted. The middle (14.51 mm) was used as a comparative reference value for full insertion in the analysis. In 3D Slicer, virtual markings were placed indicating the highest point of the abutment top, the bottom of the implant rim, and bone surface in four quadrants (Figure 1D). Per quadrant, the virtual bone surface to abutment top distance and the virtual bone surface to implant rim distance were calculated. Manual caliper measurements were obtained at every quadrant from the abutment top to the level of the artificial bone and thereafter averaged per implant. The distance of the implant rim to artificial bone was estimated by subtracting the abutment length from the caliper measurements.

#### ISQ Measurements

The Osstell ISQ (Ostell, Gothenburg, Sweden) was used to measure ISQ values by mounting a Smartpeg Type 55 on the abutments. Two perpendicular ISQ measurements (ISQ horizontal, ISQ vertical) were obtained for all implants (12). To test the influence of abutment length, ISQ values were obtained for each implant mounted consecutively with 9, 12, and 14 mm abutments, resulting in six measurements per implant. Twelve implants were tested for the high-density bone configuration, half of them fully seated and half partially seated. For the low-density bone configuration, six implants were placed, half fully seated, and half partially seated (Figure 1C). For the high-density bone configurations, one fully and one partially seated implant moved during abutment replacement. Consequently, in high-density bone, 10 BCHIs were evaluated (*n* = 5 fully seated, *n* = 5 partially seated) (Figure 1C). Due to gross implant mobility during abutment replacement, it was not possible to change the abutment in the low-density artificial bone block that was implanted with partially inserted BCHIs, hence only fully inserted BCHIs were tested (*n* = 3).

### *In Vivo* Application

After *in vitro* validation, CBCT scans were retrieved retrospectively from subjects in the outpatient clinic of Maastricht University Medical Center. The scanning protocol was identical to the *in vitro* scans. Subjects were included when a CBCT scan was performed in a clinical setting to evaluate postsurgical implant seating.

### Statistical Analysis

Statistics were performed using SPSS software (SPSS V22.0 SPSS Inc., Chicago, IL, USA). Statistical significance was established at *p* ≤ 0.05.

#### *In Vitro*—BCHI Insertion Measurements

Analyses were used for *in vitro* validation of CBCT imaging before they could be applied *in vivo*. For direct caliper measurements and CBCT measurements, mean (*M*) and SD for full insertion and partial insertion were calculated for abutment top to bone surface distances and implant rim to bone surface distances. Mean distances for abutment top to bone surface of CBCT measurements and caliper measurements for full and partial insertion were compared to each other and to the theoretical average for full insertion. Regression analysis in which the CBCT abutment top to bone surface distance and CBCT implant rim to bone surface distance were regressed to insertion depth (full or partial) was performed. 95% Prediction interval for fully inserted and partially inserted BCHIs were determined. Discriminant analysis with equal prior probabilities was applied to determine the optimal cutoff point for classification of BCHIs as fully or partially inserted.

#### *In Vitro*—ISQ Measurements

For ISQ measurements, linear mixed model analyses with implant as random factor and abutment length (9, 12, and 14 mm), artificial bone density (high, low), and insertion depth (full, partial) as fixed factors and a compound symmetry covariance matrix of the residuals. Because in the low-density artificial bone block, only fully inserted BCHIs could be measured, the effect of insertion depth could only be determined at high bone density. Similarly, the effect of bone density could only be determined for fully inserted BCHIs. When statistically significant effects were identified, Bonferroni adjusted *post hoc* comparisons between the different factor levels were additionally performed.

## Results

### *In* *Vitro*

#### BCHI Insertion Measurements

**Figure 2:**
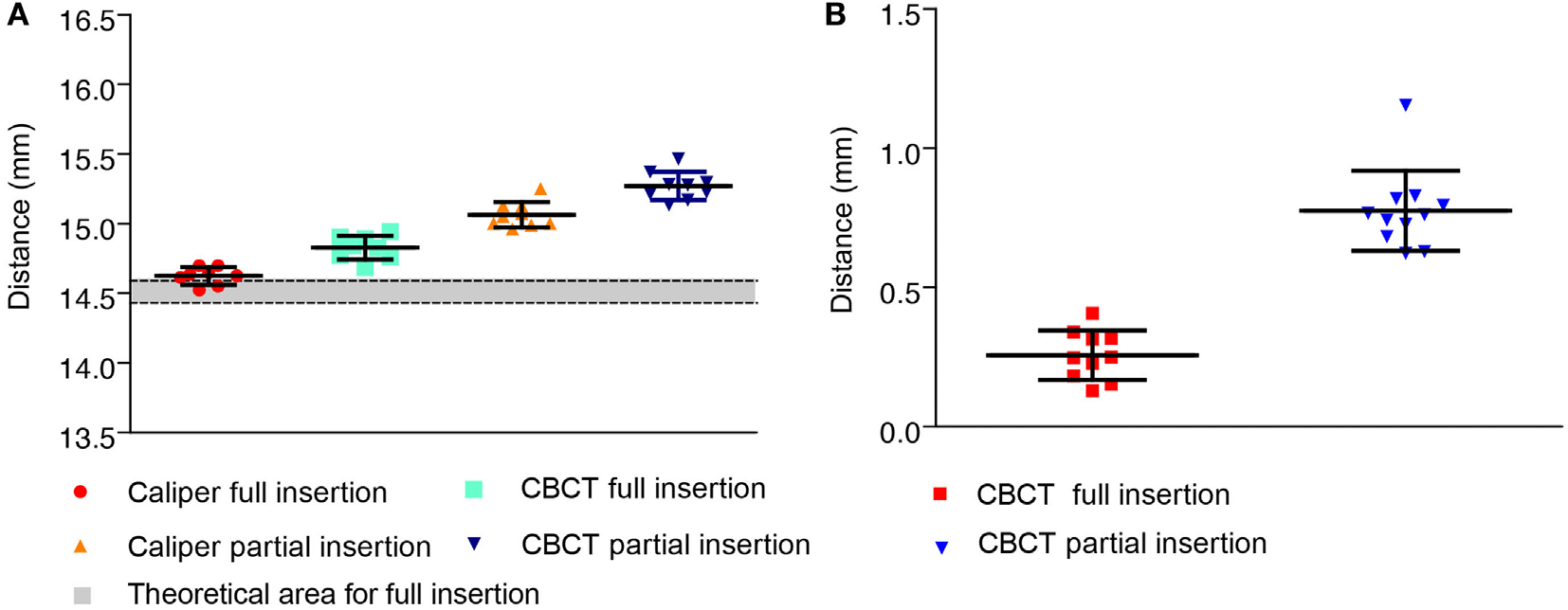
Measurements between the abutment top or implant rim and artificial bone *in vitro*. Mean (±SD) are displayed for fully (*n* = 9) and partially (*n* = 9) inserted 14 mm bone conduction hearing implants. **(A)** Distances between implant top and artificial bone surface as measured using a caliper or performed digitally on cone beam CT (CBCT) scans. The approximated theoretical area for full insertion according to the manufacturer specifications is displayed in gray. **(B)** Distances between the implant rim and artificial bone surface as virtually measured on CBCT.

Direct caliper measurements from abutment top to the bone surface of fully inserted BCHIs (*M* = 14.62, SD = 0.06, *n* = 9) were 0.11 mm greater than the theoretical average for full insertion *in vitro* (Figure 2A). CBCT measurements of fully inserted BCHIs (*M* = 14.83, SD = 0.08, *n* = 9) were on average 0.32 mm greater than the theoretical average for full insertion *in vitro*. For the partially inserted implants, direct caliper measurements (*M* = 15.06, SD = 0.09, *n* = 9) and CBCT measurements (*M* = 15.27, SD = 0.10, *n* = 9) were both 0.44 mm greater compared to the fully inserted BCHIs.

The implant rim to artificial bone surface distances as measured on CBCT were 0.47 mm larger in partially inserted BCHIs (*M* = 0.73, SD = 0.07, *n* = 9) compared to fully inserted BCHIs (*M* = 0.26, SD = 0.09, *n* = 9) (Figure 2B).

Regression analyses revealed that the type of artificial bone was a significant predictor for abutment top to bone surface distance (*p* < 0.015). 95% Prediction intervals for high density, low density, and the combined group are presented in Table 1. For implant rim to bone surface no significant difference between high and low density was found. By means of discriminant analysis, cutoff points were obtained which could potentially be used to classify BCHI’s either as fully or partially inserted (Table 1). Mean abutment top to bone surface distance for fully inserted BCHIs in high-density bone was 14.85 mm (SD = 0.06, *n* = 6) compared to 14.78 mm (SD = 0.10, *n* = 3) for fully inserted BCHIs in low-density bone resulting in a difference of 0.07 mm. Mean abutment top to bone surface distance for partially inserted BCHIs in high-density bone was 15.32 mm (SD = 0.09, *n* = 6) compared to 15.18 mm (SD = 0.05, *n* = 3) for partially inserted BCHIs in low-density bone resulting in a difference of 0.14 mm.

**Table 1:** Prediction intervals and cutoff points.

	Cone beam CT (CBCT) abutment top to bone surface distance			CBCT rim to bone surface distance		
	95% prediction interval		cut-off point	95% prediction interval		cut-off point
High, full insertion	14.67	15.04	15.09	0.04	0.39	0.47
High, partial insertion	15.14	15.50		0.56	0.91	
Low, full insertion	14.53	15.04	14.98	0.13	0.57	0.53
Low, partial insertion	14.92	15.44		0.50	0.94	
Combined, full insertion	14.63	15.03	15.05	0.07	0.44	0.49
Combined, partial insertion	15.07	15.48		0.54	0.91	

*Results for regression analysis. High, high-density bone; Low, low-density bone; Full, 4.5 rotations; Partial, 3.5 rotations*.

#### *In Vitro* 3D Models

**Figure 3:**
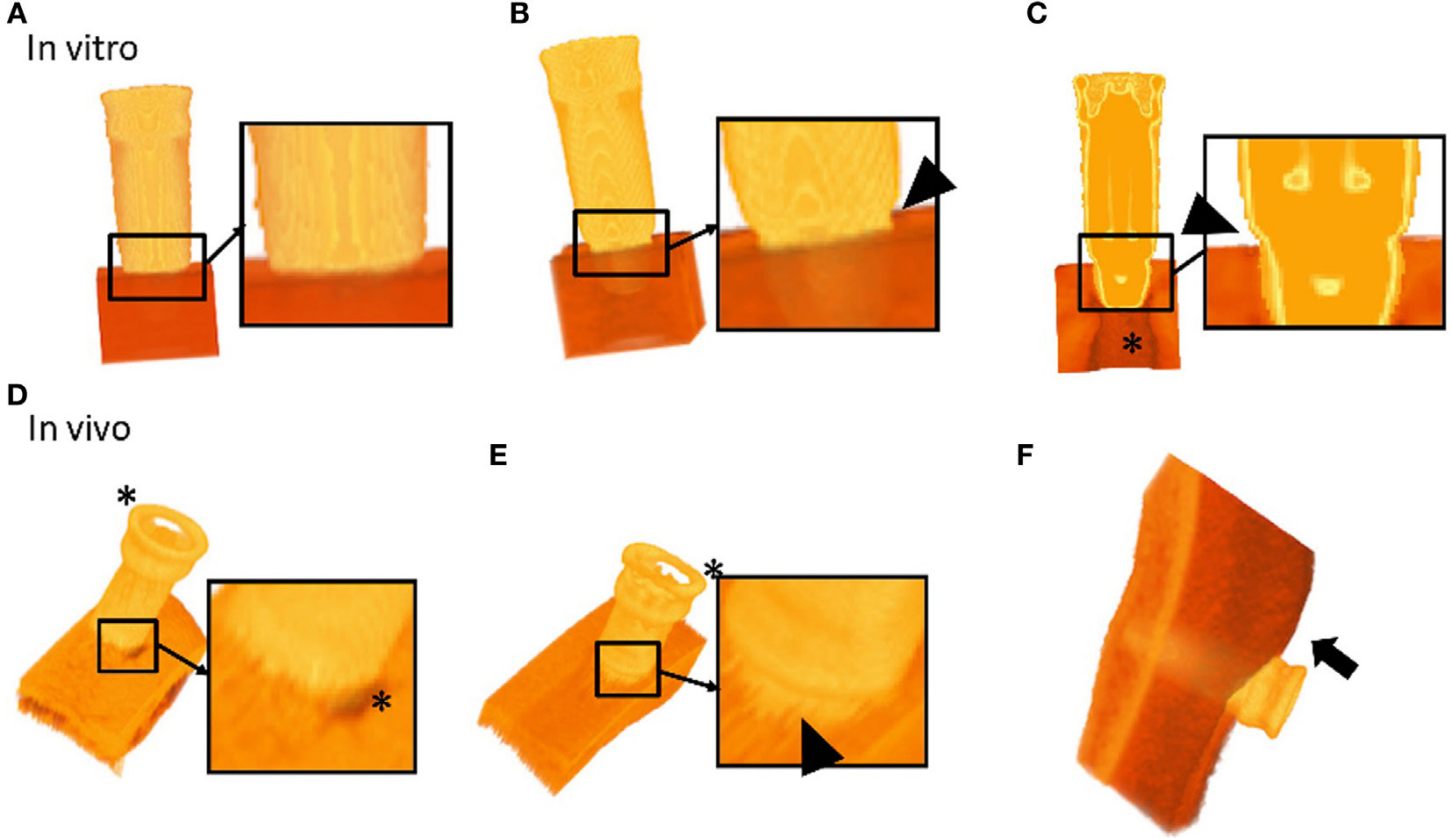
3D segmented models of bone conduction hearing implants (BCHIs) acquired by cone beam CT imaging. *In vitro* models: **(A)** 3D model of a fully inserted BCHI. Full insertion is qualitatively verified by the lack of gap between the implant rim and the artificial bone surface. **(B)** In contrast, 3D model of a bone surface is visible (arrowhead). **(C)** A cross-sectional model of a partially inserted BCHI where a gap between the implant rim and artificial bone surface is visible (arrowhead). *In vivo* models: **(D)** a 3D model of the implant–skull interface in subject 1 (full insertion). The skin was removed using a filter. **(E)** A 3D model of the implant–skull interface in subject 4 showing an angulated [full insertion on one side, distance between implant rim and surface on the opposing circumference (arrowhead)]. The skin was removed using a filter. **(F)** A 3D model of the bone, implant and soft tissue interface for subject 1. Skin sagging was present and can be discerned (arrow). Asterisks (*) indicate softening artifacts.

Evaluation of the *in vitro* 3D models allowed for a detailed qualitative assessment of the implant, artificial bone, and the implant-artificial bone interface. BCHI insertion and angulation could be evaluated as well in the axial, coronal, or sagittal plane allowing to visually distinguish between partially and fully inserted BCHIs (Figure 3). Softening effects were visible directly under the BCHI in the artificial bone models (Figure 3C).

#### ISQ Measurements

**Figure 4:**
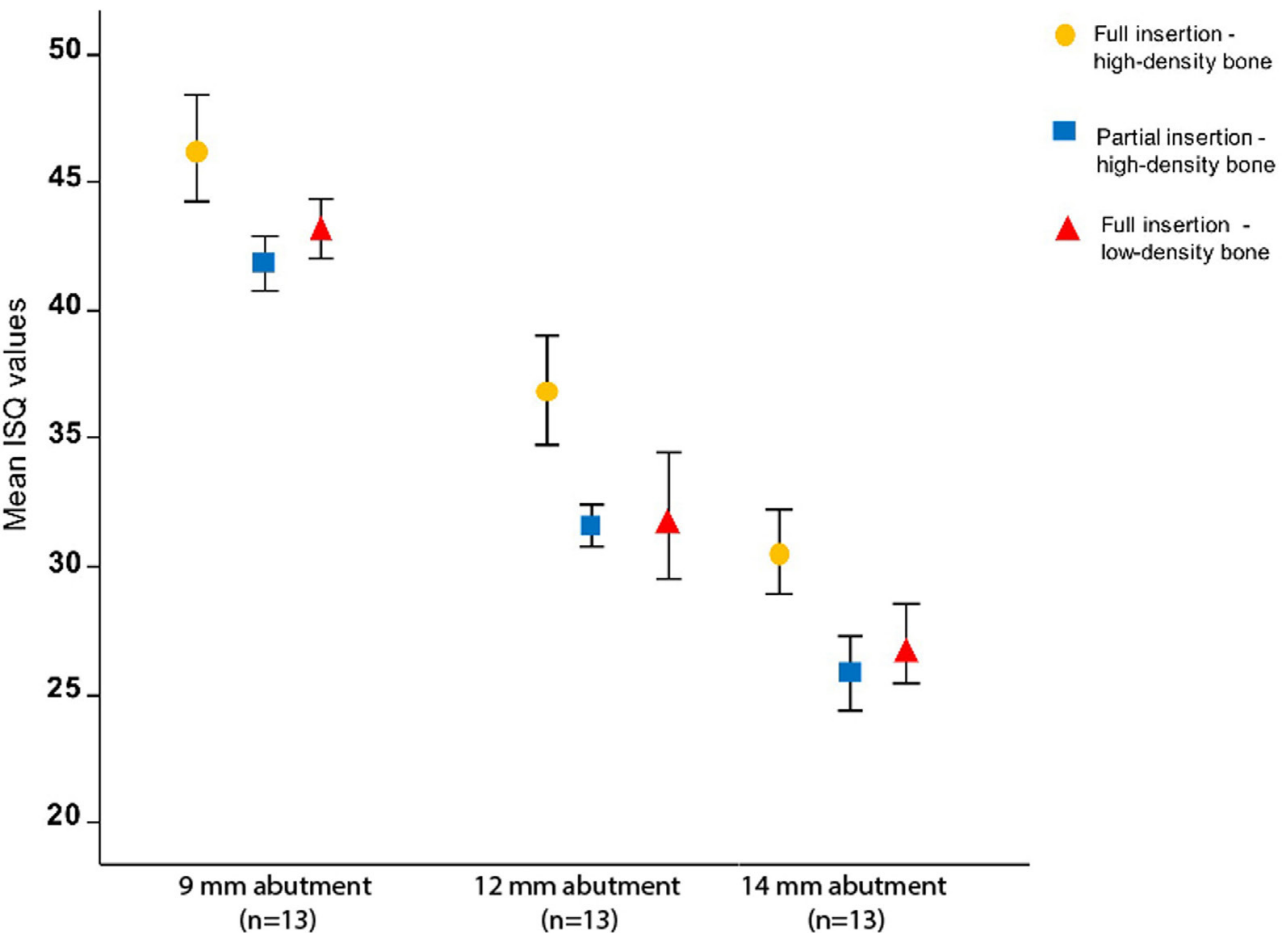
The influence of abutment length, bone density, and insertion on implant stability quotient (ISQ) *in vitro*. The mean ISQ values for high-density bone configurations (*n* = 5) and low-density bone configuration (*n* = 3) together with the SDs are plotted for different abutment lengths and artificial bone densities. ISQ values decrease significantly with increase in abutment length (*p* < 0.001), partial insertion (*p* = 0.001), and reduced bone density (*p* = 0.024). ISQ measurements of low-density partially inserted implants are missing due to an interaction between low density and partial insertion (Figure 1C).

Implant stability quotient measurements are shown in Figure 4, and the models are presented in Table 2. In a split plot ANOVA analysis, the ISQ in high-density artificial bone revealed a significant negative relationship with abutment length (*p* < 0.001) and insertion depth (*p* = 0.001), whereas no interaction between abutment length and insertion depth was found (*p* = 0.378) (Table 2). To evaluate the effect of bone density, a linear mixed model analysis was performed using the ISQ data of the fully inserted BCHIs. After removing the non-significant interaction (*p* = 0.153) between abutment length and bone density, abutment length (*p* < 0.001) and bone density (*p* = 0.024) were both significant predictors of ISQ (Table 2).

**Table 2:** Two mixed models for implant stability quotient (ISQ).

	Estimates	95% confidence interval		Degrees of Freedom	*p*
**ISQ for high-density artificial bone (*n* = 10 implants)**					
Intercept^a^	46.47	44.96	47.97	9.10	<0.001
**Insertion**					
Full (*n* = 5)	–	–	–	–	–
Partial (*n* = 5)	−4.83	−6.93	−2.73	8.00	<0.001
**Abutment length**					
9 mm (*n* = 10)	–	–	–	–	
12 mm (*n* = 10)	−9.8	−10.41	−9.19	18.00	<0.001
14 mm (*n* = 10)	−15.8	−16.41	−15.19	18.00	<0.001
**ISQ for high and low-density artificial bone (*n* = 8 implants)**					
Intercept^b^	46.58	44.62	48.54	7.36	<0.001
**Abutment length**					
9 mm (*n* = 8)	–	–	–	–	–
12 mm (*n* = 8)	−10.06	−11.04	−9.08	14.00	<0.001
14 mm (*n* = 8)	−15.88	−16.85	−14.90	14.00	<0.001
**Bone density**					
High (*n* = 5)	–	–	–	–	–
Low (*n* = 3)	−3.88	−7.05	−0.70	6.00	0.024

*Results for two mixed models. ISQ for high-density artificial bone model contains insertion depth and abutment length. Density was not used in this model because only fully inserted bone conduction hearing implants could be measured in the low-density bone blocks. ISQ for high- and low-density artificial bone model contains bone density (High/Low) and abutment length*.

*^a^Intercept indicates reference value for full insertion implant mounted with 9 mm abutment*.

*^b^Intercept indicates reference value for implant mounted with 9 mm abutment in high-density bone. – indicates reference variable*.

### *In* *Vivo*

#### Subjects

Four subjects were selected 1-week post-surgery at the ENT outpatient department of Maastricht University Medical Center to evaluate BCHI with a clinical indication. Subject characteristics are summarized in Table 3.

**Table 3:** Subject characteristics and cone beam CT (CBCT) imaging indications.

Subject	Demographics	Indication	Implant type	Surgical technique	Reason for CBCT
1	71-year-old male	Mixed hearing loss	Ponto wide implant with 12 mm abutment	Linear incision with soft tissue preservation technique	Evaluation of bone conduction hearing implant (BCHI) seating 1-week post-surgery
2	32-year-old male	Conductive hearing loss	Ponto wide implant with 9 mm abutment	Linear incision with soft tissue preservation technique	Evaluation of BCHI seating 1-week post-surgery
3	63-year-old male	Mixed hearing loss	Ponto wide implant with 14 mm abutment	Minimally invasive Ponto surgery (MIPS) technique	Evaluation of BCHI seating 1-week post-surgery
4	51-year-old female	Conductive hearing loss	Ponto wide implant with 9 mm abutment	MIPS technique	Evaluation of BCHI seating 1-week post-surgery

#### BCHI Insertion Measurements

Cone beam CT measurements are described in Table 4. Distances between the implant rim and bone surface were consistent with a full insertion (0.49 mm) for subjects 1, 2, and 3. For subject 4, the measurements per quadrant were 1.0, 0.09, 0.07, and 0.37 mm, respectively, resulting in a mean distance of 0.38 mm. The mean distance was consistent with full insertion, but the distance of 1.0 mm for one quadrant was greater than the cutoff value of 0.49 which could indicate an angulated insertion (See 3D models).

**Table 4:** Cone beam CT measurements.

Subject	Abutment length	Mean abutment top to bone distance (cutoff value)	Mean implant rim to bone distance (cutoff value)	Interpretation
1	12 mm	12.45 mm (13.05 mm)	0.06 mm (0.49 mm)	Full, straight insertion
2	9 mm	9.48 mm (10.05 mm)	0.05 mm (0.49 mm)	Full, straight insertion
3	14 mm	14.11 mm (15.05 mm)	0.16 mm (0.49 mm)	Full, straight insertion
4	9 mm	9.63 mm (10.05 mm)	0.38 mm (0.49 mm)	Full, angulated insertion^a^

*^a^Visually assessed*.

We assumed that the abutment top to bone surface cutoff distances for the 14 mm abutments could be adjusted for 9 and 12 mm abutments. The cutoff value for 14 mm abutments (15.05 mm) was therefore adapted for 9 mm abutments (10.05 mm) and 12 mm abutments (13.05 mm). Distances between abutment top and bone were consistent with full insertion in all subjects.

#### 3D Models

Evaluation of the *in vivo* 3D models allowed for qualitative assessment of the BCHI, bone, and soft tissue, allowing visual appraisal of implant seating and angulation (Figure 3). In the *in vitro* 3D models, the complete abutment could be evaluated, while softening effects around the abutment top made it difficult to evaluate the location of the abutment top in the *in vivo* 3D models. Qualitative evaluation of the post-surgery scans for subjects 1, 2, and 3 revealed a full non-angulated BCHI insertion (Figure 3D). In the postsurgery scan of subject 4, the 3D model revealed an angulated insertion of the BCHI (Figure 3E), which is consistent with the differences observed per quadrant. Unexpectedly, the 3D patient model also facilitated evaluation of the skin next to the implant. In subject 1, the presence of skin sagging, which entails excess skin superior to the abutment, was noticeable (Figure 3D).

## Discussion

Using CBCT and image analysis software, it is possible to distinguish between a normal and incomplete insertion using CBCT measurements *in vitro*. Detailed 3D models were created to evaluate BCHI seating *in vitro* and *in vivo*. In this study, we show that ISQ values are dependent on abutment length, insertion depth, and artificial bone density for Ponto implant/abutment combinations. This is consistent with other (dental) studies showing the abutment design and bone density influence (primary) stability as measured with ISQ (15).

One potential drawback with punch only surgical approaches is the lack of reference for a straight and full insertion. Surgeons may estimate implant stability using a torque wrench, but the reliability of this method is unclear. We initially tried using a small retractor to visually assess complete insertion. In our experience, this method is unreliable and may lead to undesirable tissue damage. The installation indicator developed for MIPS may be useful to detect incomplete insertion (5), but was not investigated here. It guides counting the number of rotations during BCHI installation indicating full insertion. The surgeon can manually complete the installation of the BCHI using a torque wrench in case of incomplete insertion. However, since the skull is not flat angulated seating may be missed using this approach.

### BCHI Insertion Measurements

Here, we demonstrated that CBCT imaging allows for the evaluation of implant insertion depth, seating, and angulation in an *in vitro* setting. We assume that these results could be applicable *in vivo* as well. In the *in vitro* model, the facet of the artificial bone block was flat. In the *in vivo* situation, this is however not the case due to the curvature of the scull. Nevertheless, CBCT imaging takes the curvature of the skull into account as well as demonstrated in Figures 3D,E. Although, we found statistically significant differences between bone blocks, these are relatively small. During insertion of an implant, *in vitro* or *in vivo*, the friction between the implant surface and surrounding bone will be influenced by the density, insertion torque and other properties of the bone. Hence, installing an implant with a specific insertion torque will likely result in a deeper inserted BCHI in soft compared to hard bone. Currently, new surgical techniques (5, 6), reduced time until loading of the BCHI (16), and new implants (17) are investigated. In these and other future studies, CBCT scanning techniques with image analysis might provide an opportunity for evaluation of the implant seating, implant–bone interface and possibly, temporal progression of osseointegration (8) or its decline. In this study, the diagnostic, predictive, and clinical values of CBCT imaging were not determined warranting further validation studies. Pending future validation, one might imagine its use in specific clinical indications (e.g., posttraumatic, loose implants).

### ISQ Measurements

That ISQ measurements are sensitive to a partial insertion might be explained by the different pivoting point of the implant in relation to the bone level. Hypothetically, this could lead to a different pendular movement, amplitude, and resonance frequency induced by the (constant) force exerted by the ISQ probe upon the Smartpeg. We could not change the abutments in partially inserted implants in low-density bone. How ISQ measurements should be interpreted as a clinical tool remains unclear with no validated predictive cutoff value to indicate good stability or survival. In previous studies, no correction for abutment length has been performed and usually the sample size is limited (12). Further research is necessary to investigate the multivariate role of ISQ measurements in relation to clinical outcomes.

### Limitations

This study suffers from several limitations. The mixed model results provide an estimation of the effect of abutment length, done density, and partial seating. Theoretically several interactions may play a role as well, which were not statistically significant in this model. These could relate to the small sample size of this investigation. Nonetheless, our results should be considered as approximations. CBCT measurements are known to under- or overestimate a distance (18, 19). Overestimation of CBCT measurements compared to caliper measurements was the case here as well, although minor. The measured distance can be expected to rely heavily on the type of windowing. In our experience, each CBCT scanner has different parameters for different intensities unlike the standardized Hounsfield Units. Therefore, some caution is warranted for the implementation and translation of our results. A solution to this problem would be the development of uniformly well-calibrated CBCT scanning parameters and reconstruction for this specific setup. Another limitation is the presence of hardening and softening artifacts in the area adjacent to the implant, resulting in an impaired possibility to evaluate surface directly adjacent to the BCHI. Softening effects were objectified as well directly under the implant *in vitro* (Figure 3C) and around the abutment top *in vivo*, resulting in a distorted 3D reconstruction (Figures 3D,E). Due to the softening effects of the abutment top, the reliability of the abutment top to bone surface distances should be considered lower than the implant rim to bone surface distances for these cases. Although this is unfortunate, the implant rim to bone surface distances and 3D reconstruction of the implant adjacent to the skull hold the most relevant information for determining implant seating, angulation, and implant–bone interface. The positioning of a subject in the CBCT scanner may influence softening effects of the abutment top and should be considered when performing a CBCT scan. Additionally, bone thickness adjacent to an implant might be underestimated due to softening artifacts. Further reduction of these hardening and softening artifacts could allow for better evaluation of the tissues adjacent to the BCHI enabling researchers to investigate implant stability *in vivo*.

## Conclusion

Cone beam CT imaging allows to study implant seating and insertion depth after BCHI surgery. This can be useful when visual confirmation is limited. It is possible to distinguish a partial BCHI insertion from a complete insertion in artificial bone blocks. This technique could prove to be a valuable research tool. *In vitro*, ISQ values for Ponto BCHIs relate to abutment length, insertion depth, and bone density.

## Ethics Statement

The procedures in this study were in accordance with legislation (the Medical Research Involving Human Subjects Act) and ethical standards on human experimentation in the Netherlands. CBCT scans were made to assess BCHI seating on clinical indication. According to the Medical Research Involving Human Subjects Act (WMO), ethical approval was not required due to the nature and anonymization of the data.

## Author Contributions

TC is involved in the execution and analysis of the study. MJ is involved in the design and execution of the study. MH is involved in the design and analysis of the study. TC, MH, and MJ wrote the manuscript. EB contributed to the analysis of the results. RS supervised the study. MJ is involved in the analysis of the study. All authors reviewed and edited the manuscript.

## Conflict of Interest Statement

MJ is an employee of Oticon Medical AB (Askim, Sweden). All other authors declare that the research was conducted in the absence of any commercial or financial relationships that could be construed as a potential conflict of interest.
